# Creating wounds in cell monolayers using micro-jets

**DOI:** 10.1063/5.0043312

**Published:** 2021-02-08

**Authors:** Cristian Soitu, Mirela Panea, Alfonso A. Castrejón-Pita, Peter R. Cook, Edmond J. Walsh

**Affiliations:** 1 Osney Thermofluids Institute, Department of Engineering Science, University of Oxford, Osney Mead, Oxford OX2 0ES, United Kingdom; 2 Neurosciences Group, Nuffield Department of Clinical Neurosciences, Weatherall Institute of Molecular Medicine, John Radcliffe Hospital, Oxford OX3 9DS, United Kingdom; 3 Department of Engineering Science, University of Oxford, Parks Road, Oxford OX1 3PJ, United Kingdom; 4 The Sir William Dunn School of Pathology, University of Oxford, South Parks Road, Oxford OX1 3RE, United Kingdom

## Abstract

Many wound-healing assays are used in cell biology and biomedicine; they are often labor intensive and/or require specialized and costly equipment. We describe a contactless method to create wounds with any imaginable 2D pattern in cell monolayers using the micro-jets of either media or an immiscible and biocompatible fluorocarbon (i.e., FC40). We also combine this with another method that allows automation and multiplexing using standard Petri dishes. A dish is filled with a thin film of media overlaid with FC40, and the two liquids are reshaped into an array of microchambers within minutes. Each chamber in such a grid is isolated from others by the fluid walls of FC40. Cells are now added, allowed to grow into a monolayer, and wounds are created using the microjets; then, healing is monitored by microscopy. As arrays of chambers can be made using media and Petri dishes familiar to biologists, and as dishes fit seamlessly into their incubators, microscopes, and workflows, we anticipate that this assay will find wide application in wound healing.

## INTRODUCTION

Wound-healing assays are widely used to investigate, for example, skin repair, angiogenesis, morphogenesis, and metastasis.^1–5^ Cell movements in and around wounds are complex and governed by four major interlinked parameters: cell autonomous behaviors, cell–cell interactions, matrix compositions, and concentrations of critical solutes.^6^ These are often analyzed *in vitro* by creating a wound—a cell-free area—in a confluent monolayer of cells (either by excluding cells from initially entering it or by removing cells from it) and then by tracking cells as they repopulate the cleared area.^7^

A cell-free area can be created by surrounding it with some kind of removable barrier (e.g., in the form of a solid, liquid, or gel), but this can leave residues on the substrate that may alter cell behavior during repopulation.^6^ Alternatively, the “scratch assay” is commonly applied to clear cells from a selected part of a confluent monolayer, and its versatility, simplicity, and low cost make it popular.^8^ However, it is generally performed by hand—and so often lacks reproducibility (e.g., because the application of too much force damages the substrate or underlying matrix)—and, if automated, the associated machinery can be costly. Wounds can also be created, for example, by using electrical pulses,^9^ laser ablation,^10^ or laminar flows,^11^ but these approaches have not been widely adopted, possibly due to the need for specialized equipment.

Here, we introduce a contactless method to create wounds in 2D monolayers using micro-jets. Jets of media had been used previously to detach cells locally and so quantify the strength of cell adhesion.^12,13^ We attach an aqueous or fluorocarbon micro-jet to a three-axis traverse and, thus, create wounds with almost any 2D geometry in seconds by jetting cell media or an immiscible and biocompatible fluorocarbon (FC40) onto a monolayer of cells. We then combine this with “freestyle fluidics”^14–17^ to multiplex wound production. Arrays of hundreds of chambers—each containing a wound in a monolayer of cells—can be constructed within minutes on Petri dishes, which gives the potential to screen drugs that might affect healing. As wounds with virtually any shape, size, or scale can be made using media and dishes familiar to biologists, we anticipate that this assay will find wide application in wound healing.

## RESULTS

### Principle of wounding

**FIG. 1.**
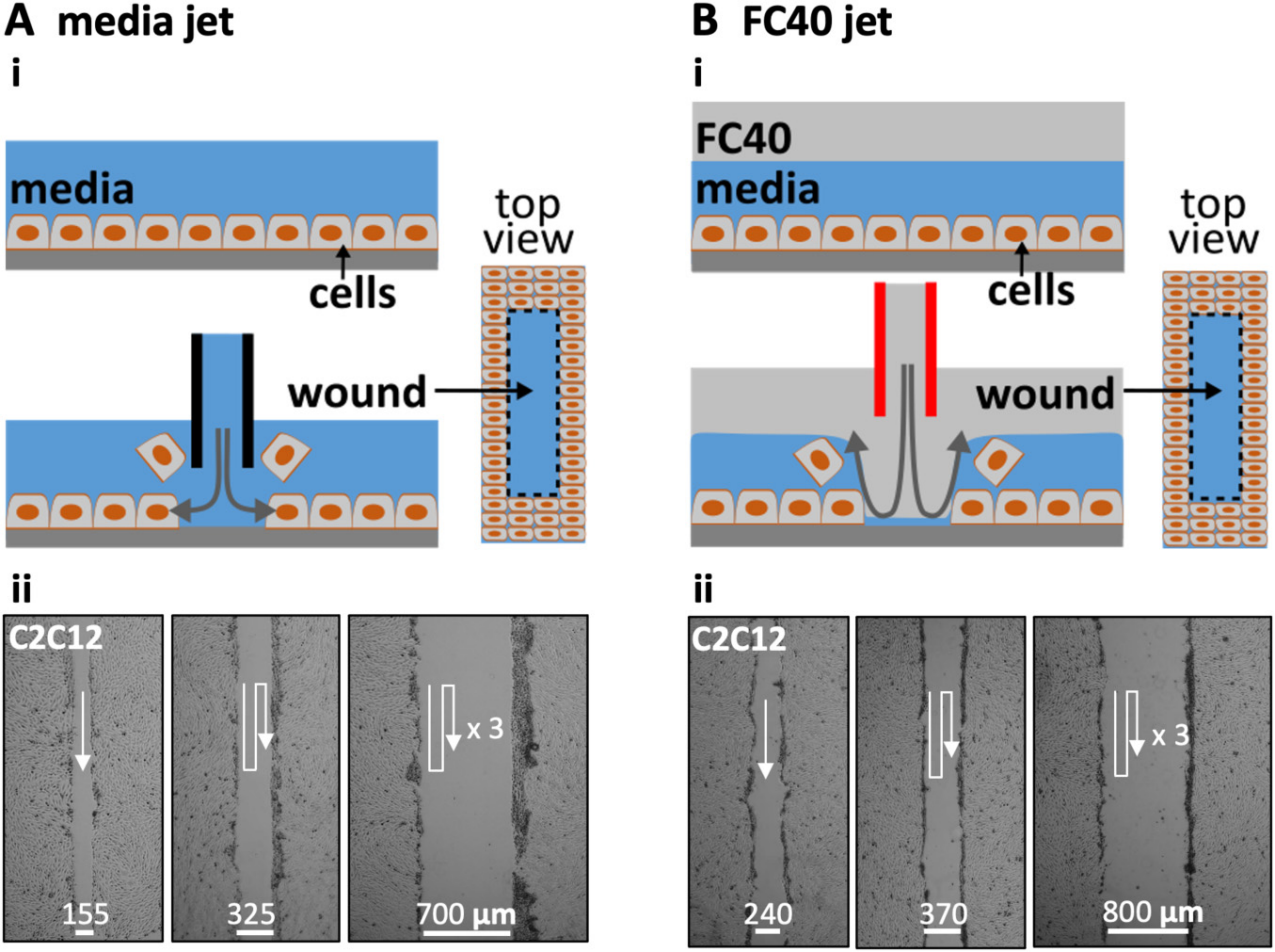
Creating wounds in a monolayer of mouse C2C12 myoblasts. (a) With media jet. (i) Cartoon showing a monolayer before and after the nozzle of a steel needle filled with media is lowered until just above the cells; on jetting media, the jet momentum detaches some cells from the surface, and moving the nozzle along a predefined path (here into the page) creates a wound (as shown in the top view). (ii) Images of wounds of different widths produced by jetting overlapping lines (white arrows: jetting paths). (b). With FC40 jet. (i) Cartoon showing a monolayer overlaid with FC40 before and after the nozzle of a steel needle filled with FC40 is lowered into the FC40 overlay until just above the media; on jetting FC40, the jet momentum forces the FC40:media interface down so that it plays on the monolayer to dislodge the cells, and moving the nozzle again creates a wound. (ii) Images of wounds with different widths created by jetting overlapping lines (white arrows: jetting paths).

Figure 1 illustrates two ways of creating wounds with micro-jets in confluent monolayers of mammalian cells. In Fig. 1(a)i, the nozzle of a stainless-steel needle (held by a three-way traverse, connected to a syringe pump, and filled with media) is lowered until 0.4 mm above the cells. When it starts jetting the media, the resulting shear stress dislodges the attached cells (Fig. S1A in the supplementary material).^13,18^ Moving the nozzle along a linear path then creates a linear wound filled with media. In practice, wounds are generated easily and quickly in monolayers of mouse C2C12 cells, with wound width being varied by overlapping jetting lines [Fig. 1(a)ii].

In the second method [Fig. 1(b)], the monolayer and media are first covered by a layer of immiscible FC40. Although the media is less dense than this overlay and one might expect buoyancy to drive it above the FC40, the media remains firmly attached to the polystyrene dish held by stronger interfacial forces. This fluorocarbon is optically transparent and freely permeable to O_2_ and CO_2_; hence, cells can be grown under it as usual in a CO_2_ incubator. The nozzle of a steel needle filled with FC40 is lowered through the fluorocarbon until it is just above the surface of the media, and the nozzle now jets FC40 instead of the media [Fig. 1(b)i]. Then, the momentum of the immiscible FC40 pushes the media aside to detach the cells, and—once the nozzle is moved—the media refills the wound (Fig. S1B in the supplementary material). As before, wound width can be varied by overlapping the jetting lines [Fig. 1(b)ii]. In this case, the bottom of the dish is pre-wetted with media (and initially covered with cells and/or materials derived from cells and serum), and the jet momentum is insufficient to sweep all the aqueous phase (and cell material) from the substrate. Consequently, the jetted FC40 does not adhere to the bottom; instead, it remains in the fluorocarbon overlay.

### Parameters affecting wound dimensions

Experimentalists often study a number of linear wounds of the same length and width. As wound length is easily controlled by starting and stopping the pump at appropriate times or places, we analyzed other parameters affecting wound width in detail, including nozzle diameter (*D_nozzle_*), height of the nozzle above the substrate (*H*), volumetric flow rate $\left(\dot{Q}\right)$, and speed of the traverse (*V_traverse_*) when making wounds (length 2.5 mm) in C2C12 myoblasts.

**FIG. 2.**
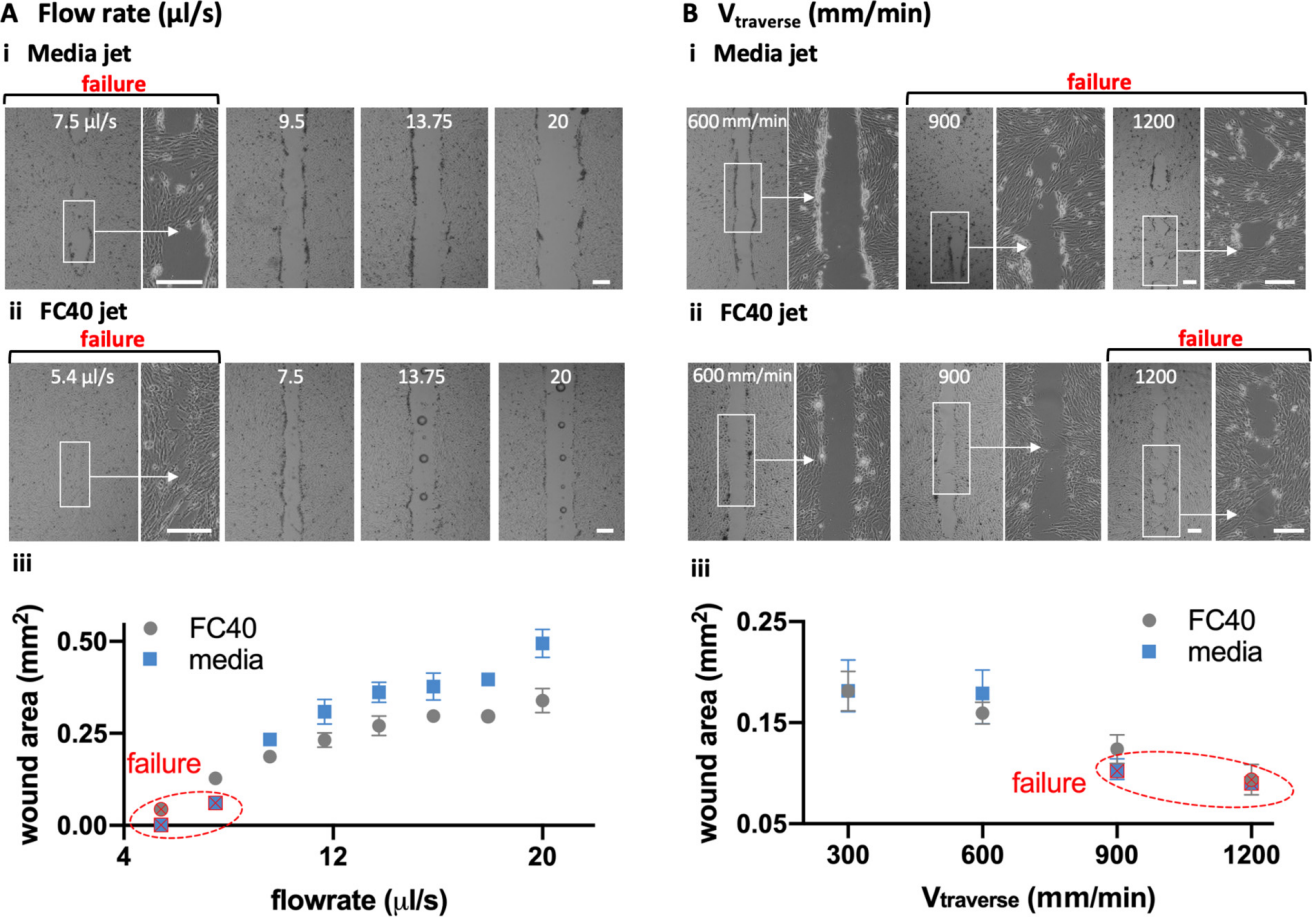
Effects of varying flow rate $\left(\dot{Q}\right)$ and traverse speed $\left(V_{traverse}\right)$ on wound width and area (*D_nozzle_ *= 60 *μ*m, *H *= 0.4 *μ*m, wound length = 2.5 mm). Scale bars: 200 *μ*m. (a). Varying $\dot{Q}$ as $V_{traverse}$ is held constant at 300 mm/min. (i) For media jets, wound failure occurs at 7.5 *μ*l/s, and increasing the flow produces wider wounds. (ii) For FC40 jets, wound failure occurs at 5.4 *μ*l/s, and increasing the flow again yields wider wounds (often with a central column of FC40 drops that adhere to the dish). (iii) Relationship between (cell-free) wound area and flow rate. Except failure conditions, each datapoint represents an average of at least n = 6 measurements. (b). Varying $V_{traverse}$ as $\dot{Q}$ is held constant at 8 *μ*l/s. (i) For a media jet, increasing the traverse rate leads to wound failure as the dwell time over a given area falls. (ii) For an FC40 jet, failure occurs at a higher traverse rate. (iii) Relationship between the cell-free wound area and the traverse rate. Except failure conditions, each datapoint represents an average of at least n = 6 measurements.

At a low media flow rate of 7.5 *μ*l/s, the jet momentum is insufficient to clear all cells off the substrate in the jetting line; we call this “failure” [Fig. 2(a)i, left]. Increasing the flow then produces progressively wider wounds [Fig. 2(a)i, right]. With an FC40 jet, failure occurs at a lower flow rate of ∼5.4 *μ*l/s, and increasing the flow again produces wider wounds [Fig. 2(a)ii]. A quantitative analysis confirms these general trends: failure occurs at low flows, increasing the flows give wider wounds, and FC40 has a lower failure point and gives narrower wounds [Fig. 2(A)iii]. Increasing the traverse rate also leads to “failure” and reduction in wound width with both media and FC40 jets [Fig. 2(b)].

Flow profiles resulting as a jet emerges from a submerged and stationary nozzle to impinge on a flat substrate are complex.^18,19^ In our case, the substrate is initially covered with cells that are removed by the jet momentum and shearing forces by the passing jet. The trends described above [Fig. 2(a)] are consistent with the jet momentum playing a critical role. For example, the longer a jet spends above a specific point, the more persistent are the shearing forces so that more cells are dislodged (resulting in wider wounds). Moreover, when the jet momentum falls below a critical value, wound production fails. In addition, FC40 is ∼1.8 times denser than media, so it gives narrower wounds, with failure occurring at a lower flow rate and higher traverse rate.

Note that the passage of an FC40 jet not only clears cells from the target area, but also leaves a column of FC40 droplets down the centerline of the wound [Fig. 2(a)ii; Soitu *et al.*,^17^ describe a related phenomenon]. This presumably arises as follows. At low flow rates, the FC40 jet cannot completely displace all media from the substrate, and FC40 never contacts polystyrene; then—as we have seen—media refills the wounded area, and the jetted FC40 rejoins the overlay as it minimizes its interfacial area with the aqueous phase. However, as the flow rate increases, the momentum becomes sufficient to sweep the media from some local areas of the substrate. Now, FC40 can contact the local areas of the dish to remain stuck there as it preferentially wets polystyrene compared to media (and so gives a column of drops). These results show that linear wounds with chosen lengths and widths can easily be made by jetting and even shapes.

Roughness of wounds is another important parameter to analyze in such applications. The smoother the wound, the easier it is to quantify cell migration into the open area. In Fig. S2 in the supplementary material, roughness of wounds made with both media and FC40 jets was compared against those made with a rod^16^ and the most widely used practice—scratching the substrate with a pipet tip. A previously published imageJ plugin^20^ was used to outline the wounds and the relative standard deviation (RSD) of the points making up the edges was used as a quantification for corrugation. Wounds made with rods and pipet tips have a similar roughness (RSDs 3.36% and 4%), whereas those created by jets produce slightly more corrugated edges (RSD 6.1% for media and RSD 6.4% for FC40). However, it was found that performing two passages with the jets over the same footprints improves the smoothness of the wound significantly, driving the RSD for media jets to 2% and 2.9% for FC40 jets.

**FIG. 3.**
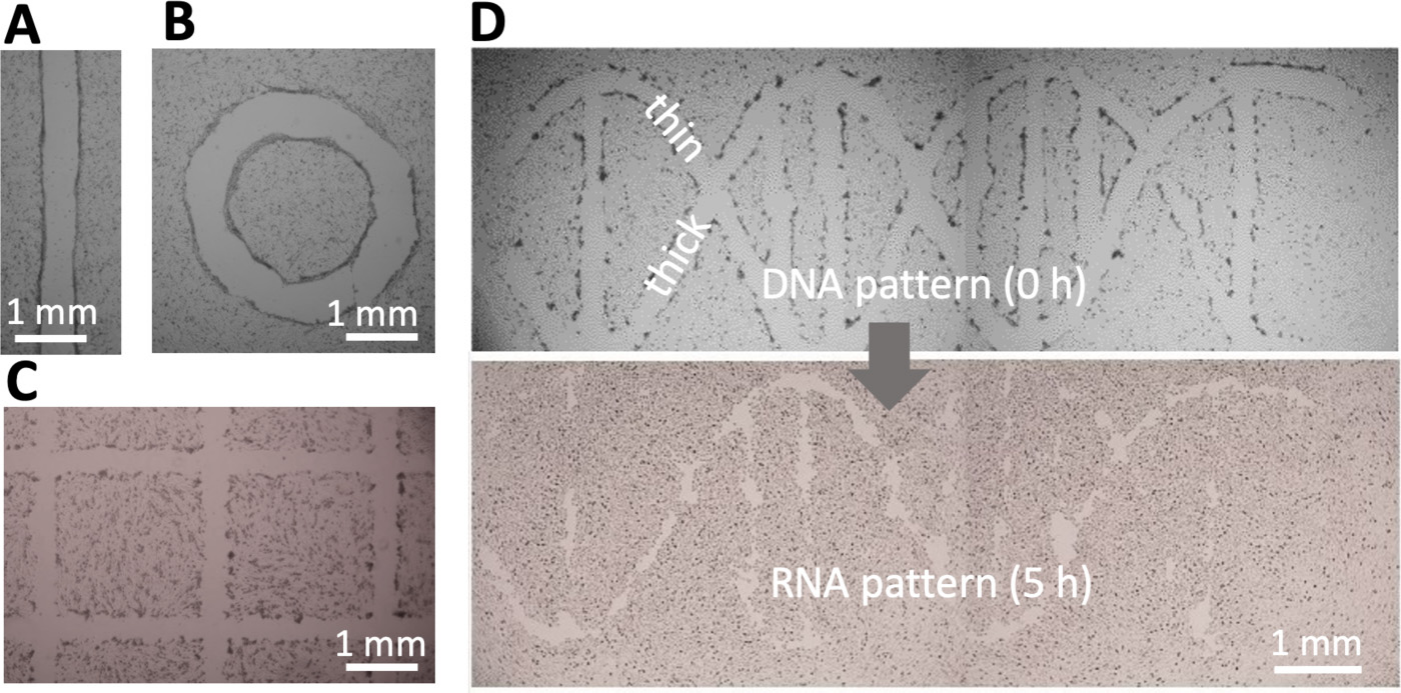
Wounding patterns. All shapes were created in 6 cm Petri dishes using media jets. (a) Line. (b) Circle. (c) A complex wound (part of 16 × 16 grid). (d) A wound shaped like a double helix of DNA (with one thick backbone strand + attached base and one thin strand + base). After incubation (5 h), the thin strand has almost completely healed, so the structure looks like a single strand of RNA.

Assay geometry is another important parameter affecting migration into a wound.^21^ Wounds with almost any imaginable 2D pattern are easily generated by altering the path followed by the micro-jet—for example, from one straight or circular line [Figs. 3(a) and 3(b)], through many straight lines in a grid [Fig. 3(c)], to two curved lines plus some straight ones [which in the upper panel of Fig. 3(d) represent the two backbone strands and base pairs in a double helix of DNA].

### Wound healing

We next illustrate healing of the wound shaped like a double helix where one helical “strand” is initially thicker than the other [Fig. 3(d), top]. Upon incubation at 37 °C, cells repopulate cleared areas to “heal” the wound. As the thin strand heals quicker than the thick one, it leaves a “single RNA strand” [Fig. 3(d), bottom]. The simpler wounds illustrated in Figs. 3(a)–3(c) also heal in <24 h (Fig. S3 in the supplementary material). These results show that wounds produced by jetting heal as expected.

**FIG. 4.**
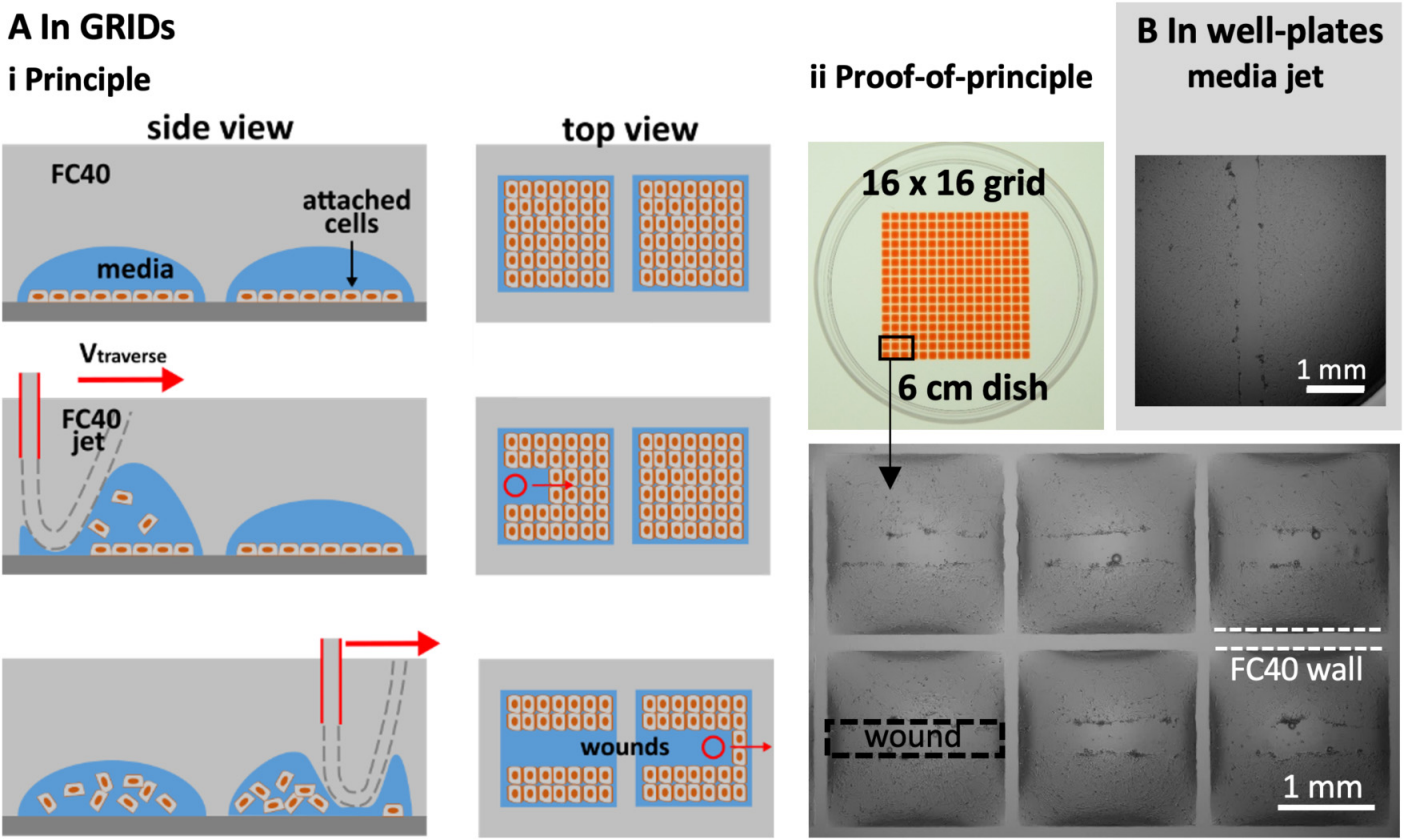
Multiplexed wounding. (a) In grids, with an FC40 jet. (i) Principle. Cells are seeded in chambers in a grid, grown to confluency, and the jet traverses above the centerline of each row; as it passes, the jet forces the FC40:media interface down so that it plays on monolayers to detach the cells. (ii) Image of grid. Chambers initially held 100 nl of media, and 400 nl of red dye is added to aid visualization. Zoom: images of wounds in six chambers. (b). In the well of a conventional 96-well plate, with a media jet.

We illustrate multiplexed wound healing using a “grid”—an array of 256 microfluidic chambers in a 6 cm dish [Fig. 4(a)].^15^ Each chamber has a square footprint of 1.9 × 1.9 mm, holds <1 *μ*l, and is isolated from others by fluid walls and ceilings of FC40. Such chambers are used much like wells in microtiter plates, albeit with one-hundredth the volume; liquids are added or removed simply by pipetting through FC40 instead of air. Then, fluid walls morph above an unchanging footprint within limits imposed by the advancing and receding contact angles around the footprint. When too much volume is added, the advancing contact angle is exceeded, the footprint expands, and neighboring chambers merge. Even so, the range of volumes that can be held before footprint expansion is greater than that held by a well in a 96-well plate. Unfortunately, the maximum volume that can be accommodated is less than the 2–6 *μ*l needed to form a long linear wound by jetting media; therefore, an FC40 jet is used.

Figure 4(a)i illustrates this principle. C2C12 myoblasts are seeded in each chamber, grown until confluent, and then the FC40 jet traverses across the middle of a chamber; the jet transiently forces the FC40:media interface down so that it plays on the monolayer to dislodge the cells, before the added FC40 returns to join the bulk FC40 in the overlay. Imaging then reveals the resulting wounds in the middle of each chamber [Fig. 4(a)ii].

Wounding can also be multiplexed using conventional 96-well plates and a media jet—as these can hold larger volumes [Fig. 4(b)]. Note the large black ring close to the well wall [Fig. 4(b)]. This “edge effect” prevents a clear view of much of the bottom of the well and is due to the solid surrounding wall obscuring the view. In contrast, edge effects in chambers with fluid walls are much less [Fig. 4(a)ii] and can be essentially eliminated using smaller volumes.^22^ These results show that our wound-healing assays can easily be multiplexed and used to screen drugs to see how they affect repopulation.

## DISCUSSION

We describe a general contactless method to create wounds in a cell monolayer; micro-jets of media or an immiscible liquid (FC40) are projected on to the monolayer to detach cells (Fig. 1, Fig. S1 in the supplementary material). We establish working conditions and failure parameters for one cell type (mouse C2C12 myoblasts) and describe ways to alter wound dimensions (Fig. 2). Wounds can have almost any imaginable 2D pattern (Fig. 3), they heal as expected [Fig. 3(d), Fig. S3 in the supplementary material], and the approach can be multiplexed (e.g., using either an FC40 jet and a 6 cm dish with 256 chambers or a media jet and a 96-well plate; Fig. 4).

Some limitations of the technology include the following: (i) A three-axis traverse and a syringe pump are essential (to produce and control the required jet). (ii) While the method is a general one, conditions have to be tuned to produce wounds with specified widths in monolayers of different cell densities and types, as both the degree of confluency and the cell type affect wound production. (iii) When using an FC40 jet, occasional FC40 drops are left behind in the wound, and these can potentially act as a physical barrier that prevents the migration of cells into all areas of the wound [Fig. 2(a)].

In summary, we have developed a contactless method to produce wounds *in vitro.* This enables experimentalists to vary the size and shape of wounds with ease. The versatility of the methods also allows them to be tuned to accommodate samples with different cell-matrix characteristics, enhancing consistency and reproducibility over multiple experiments.

## MATERIALS AND METHODS

### General reagents

All reagents and materials were purchased from Sigma Aldrich (St. Louis, Missouri), unless otherwise stated. FC40^STAR^ (iotaSciences Ltd, Oxfordshire, UK) is FC40 treated using a proprietary method that improves fluid wall formation by jetting, and throughout, we use the term ‘FC40’ to refer to it. In Fig. 4(a), Allura Red—a water-soluble dye—was added to each chamber to aid visualization.

### Cells

C2C12, an immortalized mouse myoblast cell line,^23^ was cultured in DMEM + 15% FBS. Prior to wounding, cells were plated in 60 mm tissue-culture-treated dishes (Corning, Merck product 430166) at a density of 10^6^ cells/dish and grown for ∼48 h. For Fig. 4(b), cells were plated on 96-well plates (Corning, product 3596) at a density of 15,000 cells/well and used for wounding ∼48 h later.

### Wounding and printing of grids

All jetted wounds were made using G-code and modified “isoCell” (used with 6 cm dishes) or “Pro” printers [used with 96-well plates; Fig. 4(b)] provided by iotaSciences Ltd. Each printer consists of a three-axis traverse, which moves two stainless-steel dispensing needles (Adhesive Dispensing Ltd, Milton Keynes, UK), one with a nozzle used for jetting media or FC40 (60 *μ*m ID, 0.5 mm OD) and the other to add/remove media to/from chambers (250 *μ*m ID, 0.5 mm OD). The two needles are connected to syringe pumps that control the jetting/dispensing of media/FC40.

Wounds made with the rod were also made using the Pro printer. For this, the jetting needle was replaced by a styluslike PTFE rod that was free to slide in the z direction. Now, the rod is dragged under its own weight on the bottom of the dish in a straight line, removing the cells attached to the substrate.

Grids [Fig. 4(a)] were made with DMEM plus 10% FBS. To make a grid, 1 ml of media is added to a 6 cm dish to cover the entire surface, and ∼0.9 ml is removed to leave a thin film. This is now covered with 2 ml of FC40, and the grid is created as described by Soitu *et al*. (2018).^15^ Unless otherwise stated, wounds were created using $D_{nozzle}=60\;\mu m$, *H* = 0.4 mm, $\dot{Q}=8\;\mu l/s$, and $V_{traverse}=300\;\mathrm{mm}/\min$.

The DNA-shaped wound in Fig. 3(d) was constructed after drawing the pattern in Inkscape (inkscape.org) followed by conversion to G-code, the programming language used by the printer.

### Imaging

All images of wounds were collected using a digital SLR camera (Nikon D7100 DSLR) connected to an epi-fluorescence microscope (Olympus IX53; 1.25×, 4×, 10×, 25× objectives) equipped with a translation stage and an overhead illuminator (Olympus IX3 with filters). Brightness and contrast were enhanced by 20% for these images, for better visualization of wounds. The image of the grid in Fig. 4(b) was taken using a digital SLR camera (Nikon D610). Images of wounds were analyzed using a published ImageJ (Rasband, 1997–2018) plugin.^20^ This was used to extract the areas of wounds for Fig. 2 and the borders of wounds (Fig. S2 in the supplementary material).

### Statistical analyses

The statistical analysis for Fig. 2 (averages and standard deviations) was performed in GraphPad Prism (San Diego, CA). For conditions that produced wounds successfully, each datapoint represents an average of at least n = 6 measurements. Each measurement represents the area of the surface free of cells across 1 mm long strips.

For Fig. S2 in the supplementary material, the outline of each wound was extracted using imageJ and transferred into Microsoft Excel as a collection of points with x–y coordinates. A mean was computed for each vertical border, which served in computing the standard deviation and the relative standard deviation of each point. The resulting RSDs were aggregated and transferred to GraphPad Prism for plotting. Each of the six conditions analyzed in Fig. S2 in the supplementary material contains data from at least n = 8 wounds.

## SUPPLEMENTARY MATERIAL

See the supplementary material for the physics behind the wounding process (Fig. S1), the workflow used to infer the roughness of wounds (Fig. S2), and the healing of various patterns (Fig. S3).

## Data Availability

The data that support the findings of this study are available from the corresponding author upon reasonable request.
